# Prognostic Significance of the Number of Removed and Metastatic Lymph Nodes and Lymph Node Ratio in Breast Carcinoma Patients with 1–3 Axillary Lymph Node(s) Metastasis

**DOI:** 10.5402/2011/645450

**Published:** 2011-10-12

**Authors:** Nüvit Duraker, Bakır Batı, Davut Demir, Zeynep Civelek Çaynak

**Affiliations:** ^1^Third Department of Surgery, SB Okmeydanı Training and Research Hospital, Istanbul, Turkey; ^2^Fifth Department of Surgery, SB Okmeydanı Training and Research Hospital, Istanbul, Turkey; ^3^Department of Surgery, Bayındır Levent Hospital, Istanbul, Turkey

## Abstract

We evaluated the prognostic significance of lymph node ratio (LNR), number of metastatic lymph nodes divided by number of removed nodes in 924 breast carcinoma patients with 1–3 metastatic axillary lymph node(s). The most significant LNR threshold value separating patients in low- and high-risk groups with significant survival difference was 0.20 for disease-free survival (*P* < 0.001), 0.30 for locoregional recurrence-free survival (*P* < 0.001), and 0.15 for distant metastasis-free survival (*P* < 0.001), and the patients with lower LNR had better survival. All three LNR threshold values had independent prognostic significance in Cox analysis (*P* < 0.001 for all three of them). In conclusion, LNR is a useful tool in separating breast carcinoma patients with 1–3 metastatic lymph node(s) into low- and high-risk prognostic groups.

## 1. Introduction

Axillary lymph node status is the most important prognostic factor in breast carcinoma and prognosis worsens with increasing number of metastatic lymph nodes [1, 2]. According to the American Joint Committee on Cancer (AJCC)/International Union Against Cancer (UICC) tumor (T)-node (N)-metastasis (M) classification, nodal disease is classified in three groups based on the number of axillary metastatic lymph nodes: N1, 1–3 metastatic lymph node(s), N2, 4–9 metastatic lymph nodes and N3, 10 or more metastatic lymph nodes [3]. However, the number of metastatic lymph nodes depends on the number of removed lymph nodes that are dissected by the surgeon and examined by the pathologist. Various studies have shown that the number of metastatic lymph nodes is greater with increasing number of removed lymph nodes [4–12]. It is difficult to assess the axillary lymph node status reliably without removing sufficient numbers of lymph nodes depending on the surgeon and/or pathologist.

Studies conducted in recent years indicate that the ratio of the number of metastatic lymph nodes to the number of removed lymph nodes denoted as lymph node ratio (LNR) provide a more useful prognostic information compared to nodal disease classification according to the number of metastatic lymph nodes [9, 13–16]. A review on the prognostic value of LNR indicated that the threshold value of clinically significant LNR varies in different studies and emphasized that these studies vary by sampling size and tumor stage [17]. Until now, analysis was made mostly in all lymph node-positive patients, without subdivision in N1, N2, and N3 disease groups in the LNR studies [9, 13, 14, 16, 18–23]. LNR threshold value separating the whole series in two prognostic groups with significantly different survival was given as 0.20 [18, 24] and 0.25 [9]. In three series including patients with N2 and N3 disease receiving adjuvant high-dose chemotherapy with stem-cell support, LNR threshold value of prognostic significance was determined as 0.80 by Nieto et al. [25] and Schneeweiss et al. [26], and 0.70 by Bolwell et al. [27]. Our group determined the LNR threshold value as 0.80 in a previous study including patients with T1,2,3N3M0 disease [28]. In all of the above-mentioned studies patients with higher LNR had significantly worse prognosis compared to those with lower LNR. A striking result from these studies was that the LNR threshold value of prognostic significance was greater in series excluding N1 disease, compared to those including it. Based on this, we proposed that identifying a separate LNR threshold value for each N disease group may be useful [28]. Fortin et al. recommended axillary radiotherapy for patients with a LNR 0.40 or above in the group with 1–3 metastatic lymph node(s) and for patients with a LNR 0.50 or above in the group with 4 or more metastatic lymph nodes among T1-T2 node-positive patients [7]. There is limited number of studies investigating the prognostic value of LNR in patients with only N1 disease [29–31].

In this study, we evaluated the prognostic significance of the number of removed and metastatic lymph nodes and LNR in breast carcinoma patients with 1–3 axillary metastatic lymph node(s).

## 2. Materials and Methods

### 2.1. Patients

We retrospectively reviewed the file records of women who underwent surgery for breast carcinoma between January 1993 and December 2001 and who were then followed up in SB Okmeydanı Training and Research Hospital. Inclusion criteria for the patients were a histological diagnosis of unilateral invasive breast carcinoma, no previous or concomitant malignant disease, known pathological tumor size (patients with T4 tumor were not included), axillary 1–3 lymph node(s) metastasis, no metastasis in ipsilateral internal mammary or supraclavicular lymph nodes and distant site at the time of diagnosis, microscopically tumor-free surgical margins, completion of adjuvant therapy planned according to standard therapy protocols, and a follow-up period of at least five years. A total of 924 patients (including 174 patients who underwent surgery at the study hospital) who met these criteria were enrolled in the current study. Clinicopathological and treatment features of the patients are shown in Table 1. 

Follow-up data were obtained from file records and, in some patients, through telephone calls. The endpoint of the study was disease recurrence. Locoregional recurrence was defined as the first site of recurrence involving the chest wall (local) or/and ipsilateral axillary, supraclavicular, and internal mammary lymph nodes (regional). Locoregional recurrence concurrent with distant metastasis was recorded as locoregional recurrence. First disease recurrence was recorded as distant metastasis if it was either distant metastasis or concurrent distant metastasis and locoregional recurrence. Disease-free survival (DFS), locoregional recurrence-free survival (LRFS), and distant metastasis-free survival (DMFS) times were defined as the time interval between tumor excision and detection of first disease recurrence, locoregional recurrence or distant metastasis respectively or the date of last follow-up. In 21 patients who developed a second malignancy (excluding basal cell carcinoma), the diagnosis date of the second malignancy was considered as the last followup date. In 9 patients whose death was unrelated to cancer, the date of death was considered as the last follow-up date. Fifty-five patients developed locoregional recurrence (including 16 patients who developed axillary recurrence), 243 patients developed distant metastasis, and 9 patients developed concomitant locoregional recurrence and distant metastasis; in patients without disease recurrence median follow-up time was 108.5 months.

### 2.2. Statistical Analysis

The Fisher exact test was used to compare the axillary recurrence rates of the patient groups. Patients were grouped according to the number of removed and metastatic lymph nodes and LNR threshold value. Kaplan-Meier method was used for calculation and plotting of the DFS, LRFS, and DMFS curves of the patient groups, and the log-rank test was used for the comparison of the survival curves. The relative importance of the features was investigated using the Cox proportional hazards model. All comparisons were two tailed. *P* values less than 0.05 were considered to be statistically significant. All statistical analyses were performed using SPSS version 15.0 (SPSS, Inc., Chicago, Il, USA). 

Survival analyses (for DFS, LRFS, and DMFS) were performed separately for the whole series and for the patient group having at least 10 lymph nodes removed from the axilla. Patients were grouped in two different ways according to the number of lymph nodes removed (grouping A: 1–5, 6–9, 10–15 and 16 and more removed lymph nodes; grouping B: 1–5 and 6 and more removed lymph nodes). To determine the LNR threshold value that will separate patients in two prognostic groups of low and high disease recurrence risk with significantly different survival rates, survival analyses were conducted with LNR threshold values between 0.10 and 0.40 for the whole series, and between 0.10 and 0.25 for the group with at least 10 lymph nodes removed, at increments of 0.05. The LNR that produced the significant survival difference between the groups and gave the highest log-rank *x* ^2^ value was considered as the most significant threshold value. 

## 3. Results

### 3.1. Survival according to the Number of Removed Lymph Nodes in the Whole Series

The median number of removed lymph nodes was 12 (range 1–38). 

**Figure 1:**
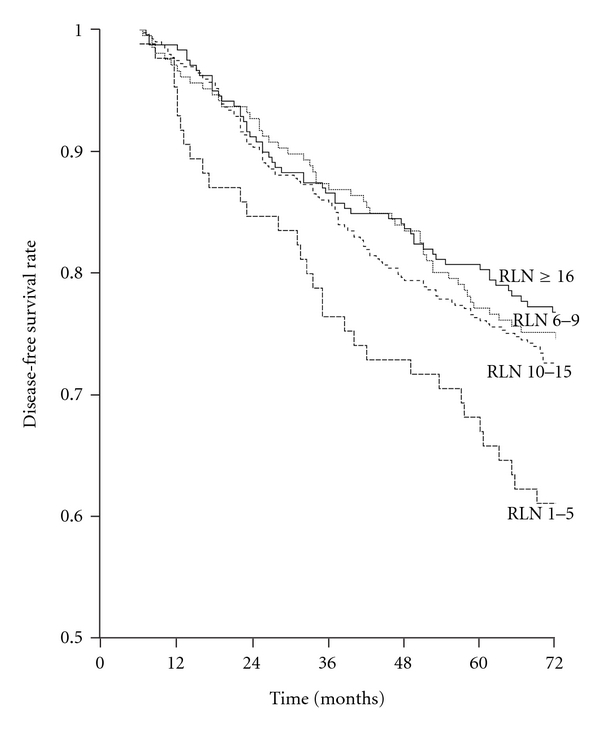
Disease-free survival rates according to the number of lymph nodes removed from the axilla. Removed lymph node(s) (RLN): 1–5 (85 patients) versus 6–9 (206 patients) (*P* = 0.016); 1–5 versus 10–15 (394 patients) (*P *= 0.024); 1–5 versus ≥ 16 (239 patients) (*P *= 0.015); 6–9 versus 10–15 (*P *= 0.703); 6–9 versus ≥ 16 (*P *= 0.985); 10–15 versus ≥ 16 (*P *= 0.593).

In grouping A, DFS was significantly worse in patients with 1–5 lymph node(s) removed compared to patients with 6–9 (*P *= 0.016), 10–15 (*P *= 0.024), and 16 and more (*P *= 0.015) lymph nodes removed; there was no significant difference between the DFS of the three groups which had 6 or more lymph nodes removed (Figure 1). In grouping B constructed based on this data, DFS of patients with 1–5 lymph node(s) removed was significantly worse than those with 6 or more lymph nodes removed (*P *= 0.008). In multivariate Cox analysis, grouping B had independent prognostic value (Table 2), whereas grouping A had not.

In grouping A, LRFS was significantly worse in patients with 1–5 lymph node(s) removed compared to patients with 6–9 (*P *= 0.008), 10–15 (*P *= 0.002) and 16 and more (*P *= 0.009) lymph nodes removed; there was no significant difference between the LRFS of the three groups which had 6 or more lymph nodes removed. According to grouping B, LRFS of patients with 1—5 lymph node(s) removed was significantly worse than patients with 6 or more lymph nodes removed (*P *= 0.001). Both groupings had independent prognostic value in Cox analysis (*P *= 0.005 and *P *< 0.001, resp.). 

In terms of DMFS there was no significant difference between patient groups according to either grouping A or B.

### 3.2. Axillary Recurrence in the Whole Series

Axillary recurrence was 9.4% (8/85 patients) in patients who had 1–5 lymph node(s) removed, while 0.9% (8/839 patients) in patients who had 6 or more lymph nodes removed, and the difference was significant (*P *< 0.001).

### 3.3. Survival according to LNR in the Whole Series

The median value of LNR was 0.143 (range 0.026–1.00) for the whole series.

**Figure 2:**
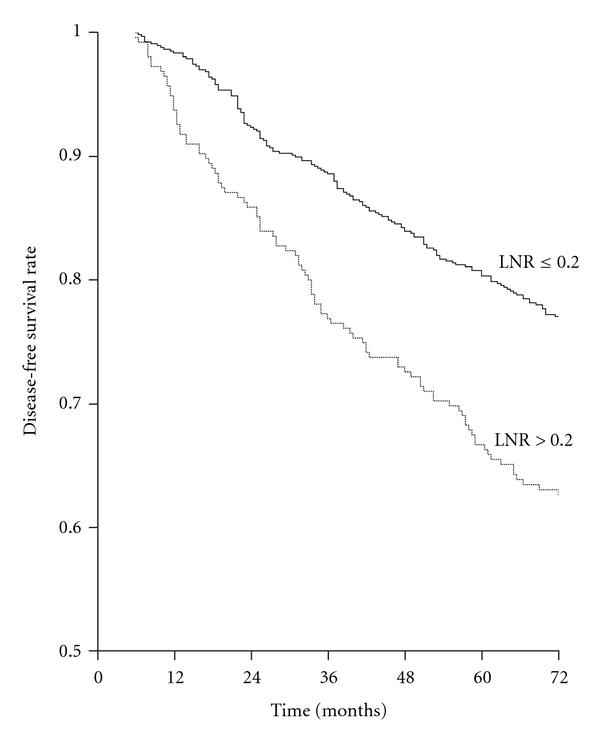
Disease-free survival rates according to lymph node ratio (LNR) in the whole series. LNR ≤ 0.20 (668 patients) versus LNR > 0.20 (256 patients) (log-rank *x* ^2^ = 19.764, *P *< 0.001).

The most significant LNR threshold value separating patients in low- and high-risk groups in terms of DFS was 0.20 (*P *< 0.001) (Figure 2); this ratio had independent prognostic significance in Cox analysis (*P *< 0.001). When grouping B, which is based on the number of lymph nodes removed and has independent prognostic significance, was added to this analysis, its prognostic significance was lost (*P *= 0.527), while the significance of LNR persisted (*P *< 0.001). 

**Figure 3:**
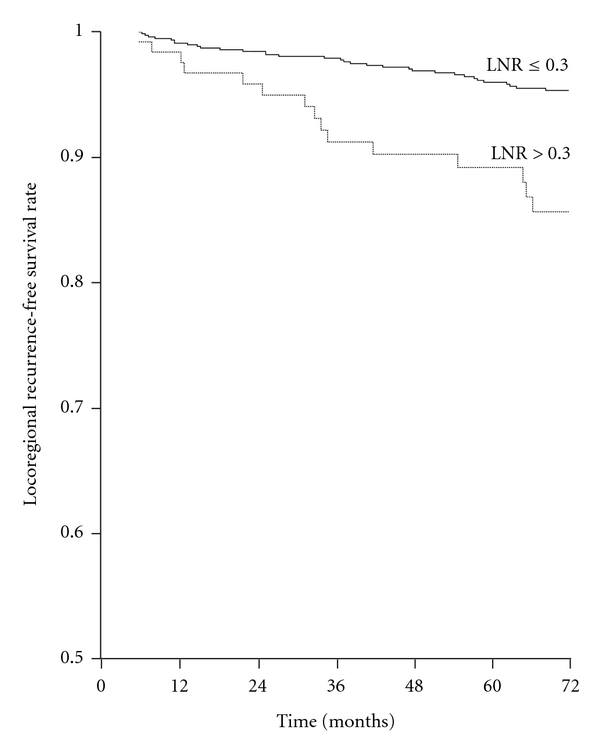
Locoregional recurrence-free survival rates according to lymph node ratio (LNR) in the whole series. LNR ≤ 0.30 (797 patients) versus LNR > 0.30 (127 patients) (log-rank *x* ^2^ = 17.611, *P *< 0.001).

The most significant LNR threshold value separating patients in two risk groups in terms of LRFS was 0.30 (*P *< 0.001) (Figure 3); this ratio had independent prognostic significance in Cox analysis (*P *< 0.001) (Table 3). When groupings A and B were added to this analysis, their prognostic significance was lost (*P *= 0.325 and *P *= 0.190, resp.), while the significance of LNR persisted (*P *= 0.004 and *P *= 0.006, resp.).

**Figure 4:**
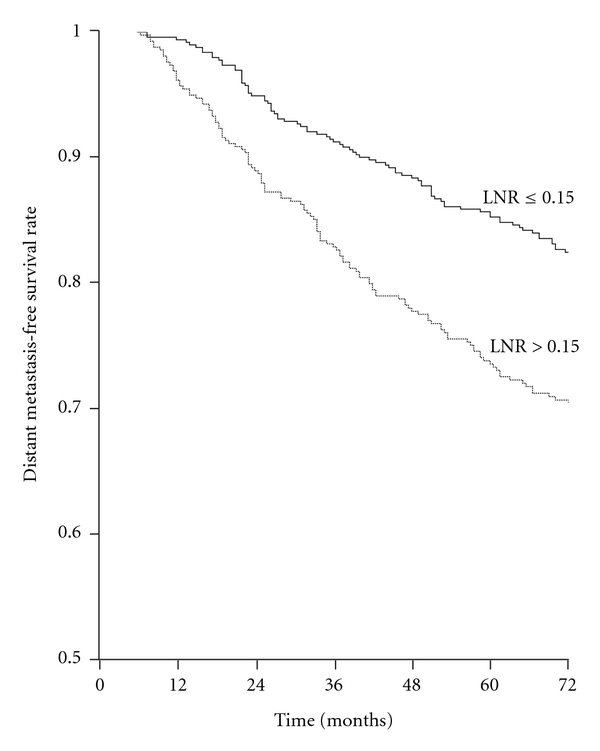
Distant metastasis-free survival rates according to lymph node ratio (LNR) in the whole series. LNR ≤ 0.15 (501 patients) versus LNR > 0.15 (423 patients) (log-rank *x* ^2^ = 17.667, *P *< 0.001).

The most significant threshold value separating patients in two prognostic groups in terms of DMFS was 0.15 (*P *< 0.001) (Figure 4); this ratio had independent prognostic significance in Cox analysis (*P *< 0.001) (Table 4).

### 3.4. Survival by the Number of Removed Lymph Nodes in Patients with at Least 10 Lymph Nodes Removed

As mentioned above, there was no significant relationship between the number of removed lymph nodes and survival based on any patient grouping for all three types of survival endpoint.

### 3.5. Survival by the Number of Metastatic Lymph Nodes in Patients with at Least 10 Lymph Nodes Removed

DFS was significantly better in patients with 1 positive node compared to those with 3 positive nodes; there was no significant difference between those with 1 and 2 positive node(s) or 2 and 3 positive nodes. Based on this, when patients were grouped as those with 1 positive node versus 2-3 positive nodes, DFS was significantly better for patients with 1 positive node (*P *= 0.016); this grouping had independent prognostic significance in Cox analysis (*P *= 0.007). 

There was no significant relationship between the number of metastatic lymph nodes and LRFS. 

**Figure 5:**
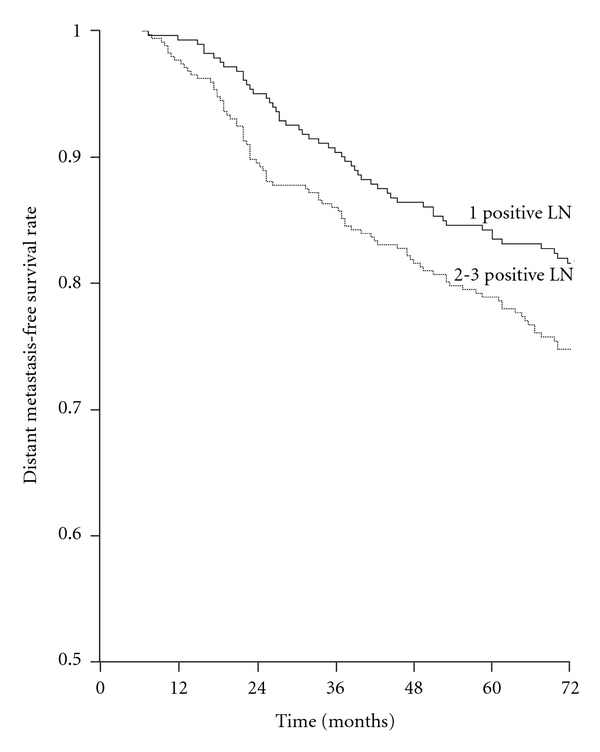
Distant metastasis-free survival rates according to the number of metastatic lymph nodes (LN) in patients with at least 10 LN removed. 1 positive (285 patients) versus 2-3 positives (348 patients) (*P *= 0.022).

DMFS of patients with 1 positive node was significantly better than those with 3 positive nodes; there was no significant difference between those with 1 and 2 positive node(s) or 2 and 3 positive nodes. Based on this, when patients were grouped as those with 1 positive node versus 2-3 positive nodes, DMFS was significantly better for patients with 1 positive node (*P *= 0.022) (Figure 5); this grouping had independent prognostic significance in Cox analysis (*P *= 0.017).

### 3.6. Survival by LNR in Patients with at Least 10 Lymph Nodes Removed

The most significant LNR threshold value to separate patients in low- and high-risk groups in terms of DFS was 0.15 (*P *= 0.005); this ratio had independent prognostic significance in Cox analysis (*P *= 0.001). When the grouping based on metastatic lymph node number (1 positive versus 2-3 positives) was added to this analysis, its prognostic significance was lost (*P *= 0.294), while the prognostic value of LNR moved slightly outside of the limit of significance (*P *= 0.064). 

There was no significant LNR threshold value to separate patients in two prognostic groups in terms of LRFS. 

**Figure 6:**
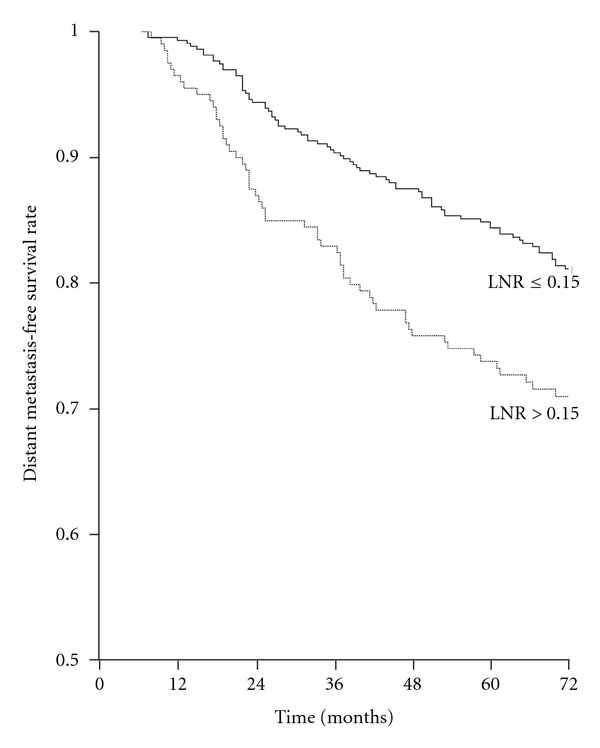
Distant metastasis-free survival rates according to lymph node ratio (LNR) in patients with at least 10 lymph nodes removed. LNR ≤ 0.15 (433 patients) versus LNR > 0.15 (200 patients) (log-rank *x* ^2^ = 12.130, *P *< 0.001).

The most significant LNR threshold value to separate patients in two prognostic groups in terms of DMFS was 0.15 (*P *< 0.001) (Figure 6); this ratio had independent prognostic significance in Cox analysis (*P *< 0.001). When the grouping based on metastatic lymph node number (1 positive versus 2-3 positives) was added to this analysis, its prognostic significance was lost (*P *= 0.803), while LNR continued to be a significant prognostic factor (*P *= 0.010). 

## 4. Discussion

In our series composed of patients with breast carcinoma having 1–3 metastatic axillary lymph node(s), DFS and LRFS of patients with 1–5 lymph node(s) removed from their axilla were significantly worse than those with 6 or more lymph nodes removed, and this grouping had independent prognostic significance for both types of survival outcomes. In patient groups with more than 5 lymph nodes removed, there was no significant relationship between the increased number of removed lymph nodes and DFS or LRFS. There was no significant relationship between the number of removed lymph nodes and DMFS.

Studies involving patients with 1–3 axillary lymph node metastasis indicate significantly better overall survival [32] and disease-free survival [33] with increasing number of lymph nodes removed. Karlsson et al. determined that in node-positive patients who did not receive radiotherapy, locoregional recurrence decreased significantly with increasing number of nonmetastatic lymph nodes removed and indicated that patients with 1–3 positive nodes and less than 10 nonmetastatic lymph nodes removed are candidates for postmastectomy radiotherapy, since their 10 year cumulative locoregional recurrence incidence is over 20% [34]. In a study by Schaapveld et al., overall survival was found to be significantly worse in patients with less than 10 lymph nodes removed compared to patients with 10 or more nodes removed; however, since overall survival can be deceptive as it includes all deaths along with nonbreast cancer related deaths, relative survival (the ratio of the overall survival and the expected survival) was analyzed and the number of removed lymph nodes was shown to be nonsignificant [8]. Truong et al. did not observe a significant relationship between the number of lymph nodes removed and locoregional recurrence, distant metastasis, and overall survival rates [29]. 

In our series, axillary recurrence rate was significantly higher in patients with 1–5 lymph node(s) removed (9.4%) compared to those with 6 or more nodes removed (0.9%). It is generally accepted that a sufficient axillary dissection to avoid leaving probable metastatic lymph nodes behind is necessary for surgical control of the disease in the axilla and for correct evaluation of the axillary status in node-positive patients. Our study results confirm this approach: high rate of axillary recurrence in patients with few lymph nodes removed indicates that actually there may be more than three metastatic lymph nodes in the axilla (N2 maybe N3 disease) and that these may have been left in the axilla. 

In our study, the most significant LNR threshold value separating patients in low- and high-risk groups with significant survival difference was 0.20 for DFS, 0.30 for LRFS, and 0.15 for DMFS. All three LNR threshold values had independent prognostic significance in Cox analysis. When grouping B (with patients grouped according to 1–5 lymph node(s) removed versus 6 or more nodes removed) which had independent prognostic significance was added to these Cox analyses conducted for DFS and LRFS, its significance was lost, while the prognostic significance of LNR threshold values persisted. Since LNR is a derivative of the number of metastatic lymph nodes and the number of lymph nodes removed, it should not be included in the Cox analysis with one of these two parameters. The prognostic significance of LNR is superior to the number of lymph nodes removed for DFS and LRFS, and LNR has independent prognostic significance for DMFS while the number of removed lymph nodes has not; thus, LNR can be used to separate patients with N1 disease in low- and high-risk groups regardless of the total number of lymph nodes removed. 

Series of patients having 1–3 positive lymph node(s) showed that patients with high LNR have worse survival compared to those with low LNR: Truong et al., in their series of patients having 1–39 node(s) removed and not receiving radiotherapy, determined the most significant LNR threshold value for locoregional recurrence, distant metastasis, and overall survival as 0.25, and recommended radiotherapy following mastectomy for patients with LNR > 0.25 [29]. Also, in another series of patients having 1–41 lymph node(s) removed and not receiving radiotherapy, Truong et al. indicated that in terms of locoregional recurrence prediction, LNR was more valuable than the number of metastatic lymph nodes and recommended postmastectomy radiotherapy for patients with LNR > 0.20, since their 10-year locoregional recurrence risk was above 20% [31]. Yildirim and Berberoglu, in their series of patients having at least 10 lymph nodes removed and not receiving radiotherapy, determined the most significant LNR threshold value for prediction of locoregional recurrence risk as 0.15 [30]. 

In our study, different threshold values for most significant LNR for DFS, LRFS, and DMFS were identified. If the survival analyses of this study were performed for DFS only, the threshold value of 0.20 would have been higher than the most significant threshold value for DMFS (0.15) and 167 patients with LNR > 0.15–0.20 would have been classified as with low risk despite their high risk for distant metastasis. Although in daily practice, it may be difficult to determine a different threshold value for each type of disease recurrence, its potential use in planning a more appropriate adjuvant therapy should be taken into consideration. 

Yildirim and Berberoglu in their series of all node-positive patients with at least 10 lymph nodes removed from the axilla, identified the optimum LNR threshold value as 0.15 for locoregional recurrence and 0.20 for distant metastasis and determined significantly higher disease recurrence rates in patients having a LNR above these thresholds [15].

In our study, survival analyses were conducted separately for patients with at least 10 lymph nodes removed. It is generally accepted that at least 10 lymph nodes need to be removed in order to classify nodal disease based on the number of metastatic lymph nodes according to TNM classification [8, 12, 35, 36]. Nodal disease classification cannot be done in patients having less than 10 lymph nodes removed, while that was the case with some of the patients in our series. When at least 10 lymph nodes are removed from the axilla and metastasis is found in 1–3 of them, these patients are classified as with N1 disease by TNM classification, and thus are accepted as a homogenous group. However, our study results suggest that N1 disease does not constitute a homogenous prognostic group: when patients were grouped according to the number of metastatic lymph nodes, DFS and DMFS were significantly better in patients with 1 positive node compared to 2-3 positive nodes and this grouping had prognostic significance independent of age, menopausal status, tumor size, histological type, adjuvant chemotherapy, radiotherapy, and hormonal therapy. Patients with LNR > 0.15 had significantly worse DFS and DMFS, and LNR had independent prognostic significance according to this threshold value in Cox analysis; when a grouping based on the number of metastatic lymph nodes was added to these analyses, its prognostic significance was lost, while the prognostic significance of LNR persisted though it moved a little outside of the limit of significance for DFS (*P *= 0.064). Moreover, as also mentioned above, LNR should be included in the Cox regression analysis alone, as it is derived from the number of removed and metastatic lymph nodes. According to these results, LNR, which is a more powerful prognostic factor than the number of metastatic lymph nodes, can be used to separate N1 disease patients having at least 10 lymph nodes removed into low- and high-risk prognostic groups for distant metastasis development, thus providing more detailed and useful prognostic information than TNM nodal disease classification. In patients having 10 or more lymph nodes removed, number of removed and metastatic lymph nodes and LNR did not have prognostic significance for LRFS. This result indirectly suggests that in N1 disease, removal of at least 10 lymph nodes may be sufficient to obtain locoregional control and removal of more lymph nodes may not be related to locoregional recurrence, and hence, N1 disease may be homogeneous in terms of locoregional control.

## 5. Conclusions

Irrespective of the number of lymph nodes removed from the axilla, LNR is a useful tool in separating breast carcinoma patients with 1–3 metastatic lymph node(s) into low- and high-risk prognostic groups. Thus, LNR may be useful to standardize adjuvant therapy for patients operated in hospitals that use different axillary dissection width and have different median number of removed lymph nodes as well as to draw reliably comparisons between treatment results coming from such different hospitals. 

## Figures and Tables

**Table 1:** Clinicopathological and treatment features of the patients.

Feature	Number of patients	%
Age, years		
Median	48	
Range	21–79	
<35	72	7.8
≥35	852	92.2
Menopausal status		
Premenopausal	494	53.5
Postmenopausal	430	46.5
Tumor size		
T1	281	30.5
T2	541	58.5
T3	102	11.0
Histological type		
Invasive ductal	789	85.3
Invasive lobular	67	7.3
Invasive ductal and lobular	31	3.4
Other	37	4.0
Surgery		
Modified radical mastectomy	828	89.6
Radical mastectomy	4	0.4
Breast-conserving	92	10.0
Adjuvant chemotherapy		
Yes	814	88.1
No	110	11.9
Adjuvant hormonal therapy		
Yes	642	69.5
No	282	30.5
Adjuvant radiotherapy		
Yes	788	85.3
No	136	14.7

**Table 2:** Cox proportional hazards model analysis of the clinicopathological features and the number of removed lymph nodes in terms of disease-free survival.

Feature	Relative risk	%95 CI	*P*
Age, years			0.028
<35	1.00		
≥35	0.65	0.45–0.95	
Menopausal status			0.277
Premenopausal	1.00		
Postmenopausal	0.86	0.66–1.12	
Tumor size			<0.001
T1	1.00		
T2	2.16	1.60–2.91	
T3	2.89	1.95–4.27	
Histological type			0.080
Invasive ductal	1.00		
Invasive lobular	1.07	0.70–1.63	
Invasive ductal and lobular	1.21	0.68–2.12	
Other	0.32	0.13–0.79	
Adjuvant chemotherapy			0.998
Yes	1.00		
No	1.00	0.66–1.50	
Adjuvant hormonal therapy			0.003
Yes	1.00		
No	1.43	1.12–1.83	
Adjuvant radiotherapy			0.001
Yes	1.00		
No	1.61	1.20–2.17	
Number of removed LN			0.022
1–5	1.00		
≥6	0.67	0.47–0.94	

**Table 3:** Cox proportional hazards model analysis of the clinicopathological features and lymph node ratio in terms of locoregional recurrence-free survival in the whole series.

Feature	Relative risk	%95 CI	*P*
Age, years			0.737
<35	1.00		
≥35	0.84	0.32–2.23	
Menopausal status			0.863
Premenopausal	1.00		
Postmenopausal	1.05	0.60–1.82	
Tumor size			0.059
T1	1.00		
T2	2.17	1.14–4.14	
T3	2.08	0.82–5.25	
Histological type			0.831
Invasive ductal	1.00		
Invasive lobular	0.95	0.37–2.42	
Invasive ductal and lobular	0.50	0.07–3.70	
Other	0.61	0.15–2.53	
Adjuvant chemotherapy			0.746
Yes	1.00		
No	0.86	0.36–2.05	
Adjuvant hormonal therapy			0.181
Yes	1.00		
No	1.44	0.84–2.47	
Adjuvant radiotherapy			<0.001
Yes	1.00		
No	3.78	2.17–6.59	
Lymph node ratio			<0.001
≤0.30	1.00		
>0.30	3.14	1.94–5.99	

**Table 4:** Cox proportional hazards model analysis of the clinicopathological features and lymph node ratio in terms of distant metastasis-free survival in the whole series.

Feature	Relative risk	%95 CI	*P*
Age, years			0.009
<35	1.00		
≥35	0.58	0.39–0.87	
Menopausal status			0.148
Premenopausal	1.00		
Postmenopausal	0.80	0.60–1.08	
Tumor size			<0.001
T1	1.00		
T2	2.13	1.53–2.97	
T3	2.94	1.92–4.51	
Histological type			0.042
Invasive ductal	1.00		
Invasive lobular	1.08	0.68–1.71	
Invasive ductal and lobular	1.50	0.85–2.64	
Other	0.24	0.07–0.75	
Adjuvant chemotherapy			0.986
Yes	1.00		
No	1.00	0.63–1.58	
Adjuvant hormonal therapy			0.016
Yes	1.00		
No	1.39	1.06–1.82	
Adjuvant radiotherapy			0.037
Yes	1.00		
No	1.47	1.02–2.12	
Lymph node ratio			<0.001
≤0.15	1.00		
>0.15	1.72	1.33–2.23	
